# Novel neuroprotective strategy based on inhibition of acute axonal palmitoylation of dual leucine-zipper kinase

**DOI:** 10.4103/NRR.NRR-D-25-00464

**Published:** 2025-09-03

**Authors:** Azita Minaei, Xiaotian Zhang, Gareth M. Thomas

**Affiliations:** Center for Neural Development and Repair, Lewis Katz School of Medicine at Temple University, Philadelphia, PA, USA; Department of Neural Sciences, Lewis Katz School of Medicine at Temple University, Philadelphia, PA, USA

The field of neurodegeneration research has long been focused on finding therapeutic strategies to effectively decrease or halt neuronal loss while minimizing side effects. A recent study titled “*Inhibiting acute, axonal DLK palmitoylation is neuroprotective and avoids deleterious effects of cell-wide DLK inhibition*”(Zhang et al., 2025), describes an innovative approach to achieve this goal. The authors target a specific post-translational modification of dual leucine-zipper kinase (DLK), palmitoylation, to selectively inhibit DLK-dependent pro-degenerative signaling and protect neurons, thereby revealing a new way to intervene and block neurodegeneration. This Perspective aims to explore the significance of these findings and propose directions for future research.

DLK is an upstream activator (a “MAP3K”) of mitogen-activated protein kinases (MAPKs) that is important for neuronal responses to axonal damage, stress, and other potentially neurodegenerative stimuli (Farley and Watkins, 2018). One key role of DLK is to control retrograde signaling from damaged/stressed axons back to neuronal nuclei, triggering activation of gene expression programs. In some situations, particularly in the adult peripheral nervous system, DLK-dependent transcription can drive axon regeneration, but often DLK-dependent retrograde signaling results in neuronal death (Jin and Zheng, 2019). Consistent with this notion, genetic knockout or knockdown, or pharmacological inhibition of DLK protects neurons from degeneration caused by a diverse array of insults, ranging from loss of trophic support to physical injury and the damaging effects of multiple toxins (Siu et al., 2018). DLK loss is also neuroprotective in several preclinical models of neurodegenerative conditions, including Parkinson’s disease, amyotrophic lateral sclerosis, Alzheimer’s disease, and damage to the optic nerve relevant to glaucoma (Siu et al., 2018). These findings, drawn from a variety of experimental systems and neuron types, identified DLK as an attractive therapeutic target. This possibility was further enhanced by the suggestion that DLK selectively mediates pro-degenerative, but not basal, physiological signaling by downstream MAPKs (Siu et al., 2018, and references therein).

Based on these promising preclinical findings, an inhibitor of the kinase activity of DLK, GDC-0134, was advanced to a clinical trial in patients with amyotrophic lateral sclerosis (Katz et al., 2022). However, some patients treated with high doses of GDC-0134 reported significant adverse effects, including symptoms of sensory neuropathy. Some patients also displayed elevated plasma levels of neurofilament-light (NFL), a biomarker of axonal cytoskeletal disruption (Katz et al., 2022). NFL was also elevated in the plasma of mice in which DLK was globally knocked out in adulthood, increasing the likelihood that effects of GDC-0134 on NFL were due to “on target” inhibition of DLK (Katz et al., 2022).

Sensory neuropathy can be caused by genetic mutations or pharmacologic agents that disrupt the cytoskeleton of dorsal root ganglion (DRG) sensory neurons, which normally convey the sense of touch and pain. Zhang et al., (2025) therefore considered the possibility that the plasma NFL elevation and neuropathy symptoms might be linked, particularly if the inhibitor that pharmacologically targeted the kinase domain of DLK also disrupted the cytoskeleton of DRG axons. Consistent with this notion, when they treated cultured DRG neurons with GNE-3511, a DLK kinase domain inhibitor structurally related to GDC-0134, they observed acute disruption of NF-200 and βIII-tubulin, core components of axonal neurofilaments and microtubules, respectively. They therefore considered ways to potentially prevent pro-degenerative retrograde signaling by DLK without triggering this cytoskeletal disruption.

Prior work had shown that pro-degenerative retrograde signaling requires DLK to be post-translationally modified with the lipid palmitate, a process known as palmitoylation or S-acylation. Notably, genetic mutation of the palmitoylation site of DLK can protect neurons from degeneration as effectively as complete DLK loss or pharmacological inhibition, both in vitro and in vivo (reviewed in (Zhang and Thomas, 2024)). However, these prior studies could not distinguish between a role for basal, constitutive palmitoylation of DLK *versus* stimulus-dependent palmitoylation of DLK in axons. In their recent study, Zhang et al. (2025) showed that DLK is indeed acutely palmitoylated in distal axons in response to the stress of trophic factor deprivation. Moreover, consistent with another recent report (Tortosa et al., 2022), this palmitoylation event coincides with increased recruitment of DLK to axonal vesicles, which likely convey retrograde signals. This finding suggests the existence of distinct pools of DLK — one regulated under basal conditions and another activated in response to stress. By selectively targeting the stimulus-dependent pool, Zhang et al. (2025) hypothesized that it might be possible to inhibit pro-degenerative DLK signaling without inducing cytoskeletal disruption.

**Figure 1:**
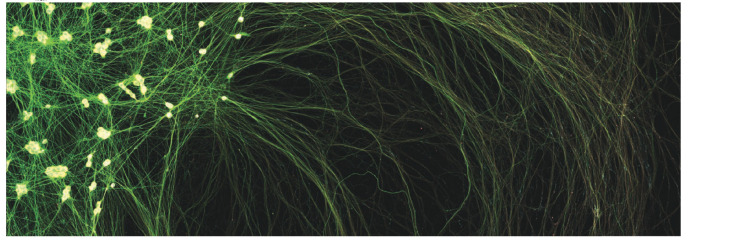
Image of DRG neurons grown in a “spot” culture, as used by Zhang et al. (2025) to selectively isolate material from DRG axons. Loss of trophic factor support or other forms of axonal stress and damage can trigger DLK-dependent axon-to-soma signals in these cultures and subsequent degeneration. Using these cultures and other systems, Zhang et al. (2025) revealed a new strategy to prevent such degeneration, by identifying compounds that selectively block the stimulus-dependent palmitoylation of DLK. The image shows a DRG spot culture immunostained to detect neurofilament-200 (green), DLK (red) and tuj1 (cyan). Unpublished data. DRG: Dorsal root ganglion; DLK: dual leucine-zipper kinase.

To test this hypothesis, Zhang et al. (2025) expanded a high-content imaging approach to screen over 28,000 compounds, seeking those that disrupt the palmitoylation-dependent subcellular localization of DLK. This strategy resulted in 33 candidate compounds, two of which, referred to as compounds **8** and **13**, demonstrated remarkable neuroprotective properties in cultured DRG neurons subjected to the stress of trophic factor deprivation. By combining conventional DRG cultures with a ‘spot’ culture system that allowed selective harvesting of axonal material (**Figure 1**), the authors showed that their compounds selectively inhibited the acute, axonal palmitoylation of DLK and subsequent retrograde signaling, without affecting basal DLK signaling. Both **8** and **13** also protected cultured DRG neurons from neurodegeneration caused by prolonged trophic factor deprivation and, importantly, neither compound induced the cytoskeletal disruption observed with the DLK kinase domain inhibitor (Zhang et al., 2025). Moreover, these novel compounds were not only protective in cultured DRG neurons but also effectively inhibited DLK signaling following optic nerve crush. Optic nerve crush is a well-established axonal injury model in which DLK-dependent retrograde signaling activates a transcriptional response in retinal ganglion cell bodies that ultimately leads to neurodegeneration. Specifically, intravitreal injection of either **8** or **13** significantly reduced DLK-mediated phosphorylation of c-Jun in retinal ganglion cells after optic nerve crush. This result indicates that **8** and **13** can reduce DLK-dependent retrograde stress signaling *in vivo*, thereby highlighting the therapeutic potential of these compounds.

**Figure 2:**
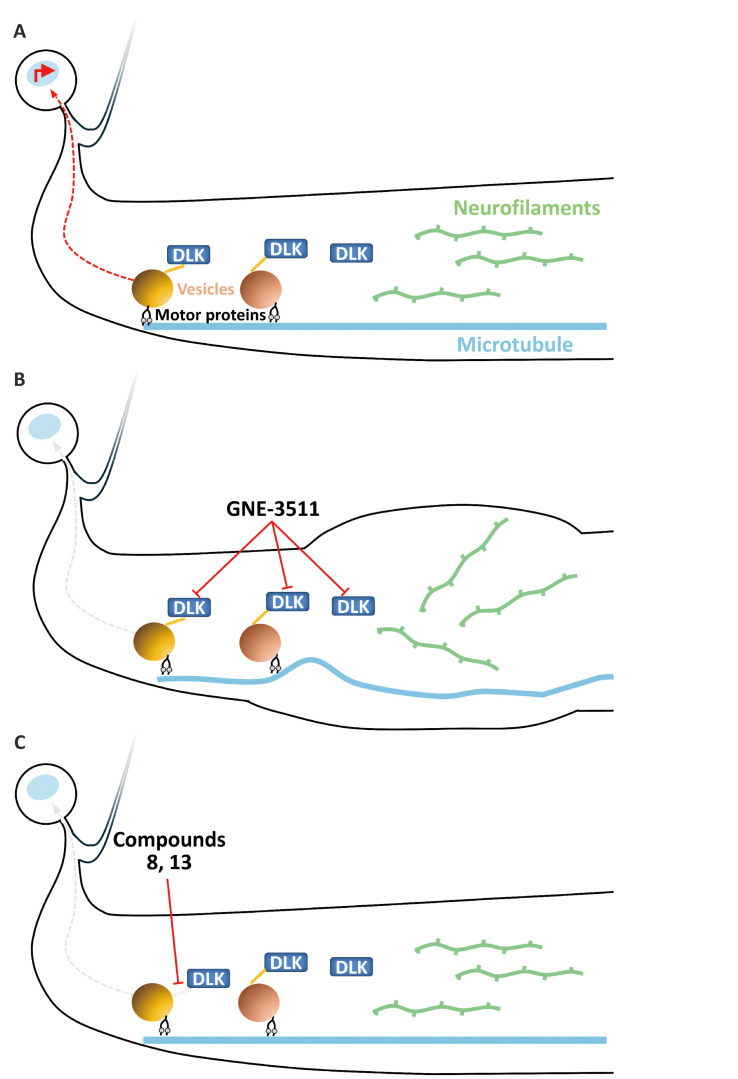
Summary of key findings from Zhang et al. (2025). (A) After trophic factor deprivation, and likely after other forms of axonal damage and stress, a pool of dual leucine-zipper kinase (DLK) is acutely palmitoylated (indicated by gold zigzag), triggering attachment to vesicles and axon-to-soma signaling (red dotted line) and a transcriptional response in the cell body. A separate pool of DLK is already palmitoylated and vesicle attached under basal conditions. There is likely also a pool of DLK that remains unpalmitoylated. (B) The compound GNE-3511 inhibits the kinase domain of DLK and blocks all cellular signaling by DLK. GNE-3511 prevents DLK-dependent axon-to-soma signaling but also triggers disruption of the axonal cytoskeleton and vesicle-based transport. Whether these latter effects are due to on-target action of GNE-3511 is not yet known. (C) Compounds **8** and **13** were identified in a High Content Screen for molecules that disrupt the palmitoylation-dependent localization of DLK. In neurons, **8** and **13** selectively inhibit stimulus-dependent palmitoylation of DLK, and subsequent axon-to-soma signaling, but leave the axonal cytoskeleton intact. Adapted from Zhang et al. (2025).

By focusing on a specific post-translational modification and subcellular pool of DLK, the authors revealed a strategy to selectively and effectively inhibit pro-degenerative retrograde signaling, an approach that could potentially be used to develop new therapies for acute injury and chronic neurodegenerative diseases. A schematic summarizing the findings of the study by Zhang et al. (2025) is shown in **Figure 2**.

Despite these striking findings, important questions remain unanswered regarding therapeutic targeting of DLK. Zhang et al. (2025) acknowledge that it is unclear to what extent on- *versus* off-target action of DLK kinase domain inhibitors accounts for effects of these compounds on the axonal cytoskeleton. The elevated plasma NFL in DLK (*Map3k12*) knockout mice renders the former possibility more likely but needs to be investigated. If deleterious effects of GNE-3511 and/or GDC-0134 are due to off-target activity, a key question is whether other DLK kinase domain inhibitors with different scaffolds, which would likely not share the same off-targets, might still be promising therapeutics.

A broader question is whether approaches similar to those used by Zhang et al. (2025) could be used to develop novel therapeutics for additional neuropathological conditions. The cell-based high-content screening (HCS) assay that they employed has both advantages and disadvantages in drug discovery and development. One key strength of such assays is their ability to identify compounds that affect specific, and potentially complex, cellular phenotypes (Michelini et al., 2010). Conversely, though, hit compounds that disrupt such “endpoint” phenotypes are sometimes identified without precise knowledge of the mechanism of action. For example, Zhang et al. (2025) used the subcellular localization of DLK in human non-neuronal cells as a proxy for palmitoylation, a readout that could not distinguish between compounds that affect basal versus stimulus-dependent palmitoylation of DLK. Nonetheless, the authors met their goal of identifying compounds that selectively prevent axonal, stimulus-dependent palmitoylation of DLK and which were highly neuroprotective. Another advantage of Zhang et al. (2025) ’s screen was its use of a cotransfected mCherry marker as an internal cut-off to exclude compounds that are cytotoxic or that broadly inhibit transcription or translation. Such screens can thus filter out compounds that would otherwise show promise in pure in vitro screening assays, but which then fail in later stages of clinical development due to unappreciated toxicity in human cells.

If HCS approaches were to be more broadly employed, the assays and readouts used need not be limited to studies of neurodegeneration and indeed are equally suited to identifying compounds that affect an array of cellular phenotypes. For example, neurite outgrowth is a readily quantifiable, HCS-compatible metric, with compounds that accelerate this process being of particular interest to those focused on mechanisms of neural regeneration. Another HCS-compatible metric is cell size, which is often increased by inappropriate activation of the mechanistic target of rapamycin (mTOR) signaling pathway, resulting in a set of neurodevelopmental disorders broadly termed mTORopathies. HCS could identify compounds that ameliorate hypertrophy in cellular models of unchecked mTOR signaling, even if the mechanism by which such hypertrophy is reduced remains unknown. HCS also need not be performed with small molecule libraries; shRNA-and CRISPR interference-based HCS has successfully identified genes that control synapse number and other aspects of neuronal morphology and function (Sharma et al., 2013; Tian et al., 2021).

The study by Zhang et al. (2025) thus not only reveals a novel strategy to inhibit DLK-dependent signaling but also exemplifies the power of cell-based screening approaches to develop neuroprotective therapeutics. Future research should aim to further optimize these compounds to improve their potency and/or drug-like properties and to test their efficacy in a broader range of neurodegenerative conditions.

*We thank Natasha Hesketh (Thomas Lab, Lewis Katz School of Medicine at Temple University, USA) for assistance with Figure preparation.*

*This work was supported by NIH (R01 NS094402 and R21 EY029386) Shriners Children’s (#85190 PHI and #87400 PHI) and by BrightFocus Foundation (G2019267) (to GMT).*

*A Patent Application No. 16/631,969 (National Stage Application of PCT/US18/42620) related to the screening method used in the manuscript that is highlighted in this Perspective was jointly filed by Temple University and Shriners Hospitals for Children. GMT (inventor) is named in the patent application. The patent application is being overseen by Temple University in accordance with its appropriate policies. The remaining authors declare no conflicts of interest.*

**Additional file:** *Open peer review report 1.*

OPEN PEER REVIEW REPORT 1
