# Supplementary material for: Novel neuroprotective strategy based on inhibition of acute axonal palmitoylation of dual leucine-zipper kinase
Source: Neural Regen Res. 2025 Sep 3;21(8):3515–6. doi: 10.4103/NRR.NRR-D-25-00464 (PMC13452656; doi:10.4103/NRR.NRR-D-25-00464)
Supplement: Supplementary file 1 [file NRR-21-3515_Suppl1.pdf]

OPEN PEER REVIEW REPORT 1

Name of journal: Neural Regeneration Research
Manuscript NO: NRR-D-25-00464
Title: Novel neuroprotective strategy based on inhibition of acute, axonal palmitoylation of DLK
Reviewer's Name: Rebeca Mejias Estévez
Reviewer's country: Spain

COMMENTS TO AUTHORS

The manuscript is a commentary on another study. I believe the study is discussed in a clear and organized manner, analyzing its advantages and disadvantages, as well as its usefulness in the field. The text is clear and well-organized. However, some minor changes are suggested:

- Some defined abbreviations are not used later so that I would remove them. For example, KO'd on line 11 of page 4 or MoA on line 21 of page 6.
- In line 36 of page 5, results from a model of CNS injury are mentioned. I would add a little more detail about the results mentioned, as the discussion of these particular results is too general.
- In line 54 of page 5, there is a typo before reference number 7. Please review the sentence, as "and in" should be deleted or the sentence modified in another way because it is not correct as it is.
